# Draft Genome Sequences of Five Multilocus Sequence Types of Nonencapsulated *Streptococcus pneumoniae*

**DOI:** 10.1128/genomeA.00520-13

**Published:** 2013-07-25

**Authors:** Lance E. Keller, Jonathan C. Thomas, Xiao Luo, Moon H. Nahm, Larry S. McDaniel, D. Ashley Robinson

**Affiliations:** Department of Microbiology, University of Mississippi Medical Center, Jackson, Mississippi, USAa; Department of Medicine, University of Mississippi Medical Center, Jackson, Mississippi, USAb; Department of Surgery, University of Mississippi Medical Center, Jackson, Mississippi, USAc; Department of Pathology, University of Alabama at Birmingham, Birmingham, Alabama, USAd; Department of Microbiology, University of Alabama at Birmingham, Birmingham, Alabama, USAe

## Abstract

Nonencapsulated *Streptococcus pneumoniae* can colonize the human nasopharynx and cause conjunctivitis and otitis media. Different deletions in the capsular polysaccharide biosynthesis locus and different multilocus sequence types have been described for nonencapsulated strains. Draft genome sequences were generated to provide insight into the genomic diversity of these strains.

## GENOME ANNOUNCEMENT

Colonization of the human nasopharynx by *Streptococcus pneumoniae* is a prerequisite for pneumococcal pneumonia, meningitis, and otitis media (1, 2). More than 50% of children under five years old in the United States have been colonized by *S. pneumoniae* (3, 4). The incidence of invasive pneumococcal disease (IPD) has significantly decreased since the introduction of conjugate vaccines based on seven, and more recently, 13 capsular polysaccharides (5, 6). However, the incidences of carriage and IPD have increased for some capsular serotypes not covered by the conjugate vaccines (7, 8), and the incidence of carriage has increased for nonencapsulated and nontypeable pneumococci (9, 10).

Some nonencapsulated strains possess nonsense mutations in the capsular polysaccharide biosynthesis locus, *cps* (11). However, Park et al. (12) identified two structural variations in nonencapsulated *S. pneumoniae* called null capsule clades (NCCs), which are caused by deletions of *cps*. In one of these variations, the *cps* genes are replaced by the gene for pneumococcal surface protein K, which is involved in murine nasal colonization (12, 13). Using multilocus sequence typing, nonencapsulated strains are found to belong to different sequence types (STs) and different clonal complexes, suggesting that they are not all closely related (12).

Five nonencapsulated strains of *S. pneumoniae*, representing different *cps* deletions and STs, were selected for whole-genome sequencing: *S. pneumoniae* MNZ11b, a subline of MNZ11 (12), and *S. pneumoniae* MNZ37 (NCC1; ST8966 and ST1106); *S. pneumoniae* MNZ14 and MNZ85 (NCC2a; ST448 and ST2315); and *S. pneumoniae* MNZ41 (NCC2b; ST6153). Genomic DNA was isolated with a DNeasy kit (Qiagen), and double-stranded DNA (dsDNA) was quantified with a Qubit fluorometric assay (Invitrogen). Multiplexed paired-end libraries (2 × 150 bp) were prepared using a Nextera XT DNA sample preparation kit (Illumina). Sequencing was done with an Illumina MiSeq. CLC Genomics Workbench v6.0 software was used for quality trimming of the reads and *de novo* assembly.

An average of 7.86 million paired-end reads per strain was collected. The number of scaffolds, scaffold N_50_, and total sequence length, respectively, for each strain were as follows: 54, 73.44 kb, and 2.02 Mb for MNZ11b; 72, 61.10 kb, and 2.13 Mb for MNZ14; 66, 50.17 kb, and 1.95 Mb for MNZ37; 97, 38.75 kb, and 2.13 Mb for MNZ41; and 97, 41.84 kb, and 2.15 Mb for MNZ85. Each scaffold from each strain had an average coverage of >146×. Open reading frames were identified and annotated with the NCBI Prokaryotic Genomes Automatic Annotation Pipeline (14). The number of putative protein-coding genes and tRNAs, respectively, detected for each strain were as follows: 2,090 and 29 for MNZ11b, 2,167 and 33 for MNZ14, 1,997 and 31 for MNZ37, 2,185 and 27 for MNZ41, and 2,190 and 15 for MNZ85. The 5S, 16S, and 23S rRNA genes for each strain were assembled into a single contig, so consensus sequences for these genes were reported.

These genome sequences represent a diverse sample of nonencapsulated pneumococci. Previously reported deletions of *cps* (12) are confirmed by these sequences. This additional sequence information may aid future vaccine designs that may become necessary as nonencapsulated and other pneumococcal populations respond to the ecological effects of the current conjugate vaccines.

### Nucleotide sequence accession numbers.

The GenBank accession no. for version 1 of these sequences are ASJF00000000, ASJO00000000, ASJP00000000, ASJQ00000000, and ASJW00000000 for strains MNZ85, MNZ14, MNZ37, MNZ41, and MNZ11b, respectively.

## References

[1] Bogaert D De Groot R Hermans PW 2004 *Streptococcus pneumoniae* colonisation: the key to pneumococcal disease. Lancet Infect. Dis. 4:144–154 14998500 10.1016/S1473-3099(04)00938-7

[2] Simell B Auranen K Käyhty H Goldblatt D Dagan R O’Brien KL Pneumococcal Carriage Group 2012 The fundamental link between pneumococcal carriage and disease. Expert Rev. Vaccines 11:841–855 22913260 10.1586/erv.12.53

[3] Gray BM Converse GM Dillon HC 1980 Epidemiologic studies of *Streptococcus pneumoniae* in infants: acquisition, carriage, and infection during the first 24 months of life. J. Infect. Dis. 142:923–933 7462701 10.1093/infdis/142.6.923

[4] Black S Shinefield H 1997 Issues and challenges: pneumococcal vaccination in pediatrics. Pediatr. Ann. 26:355–360 9188126 10.3928/0090-4481-19970601-04

[5] Poehling KA Talbot TR Griffin MR Craig AS Whitney CG Zell E Lexau CA Thomas AR Harrison LH Reingold AL Hadler JL Farley MM Anderson BJ Schaffner W 2006 Invasive pneumococcal disease among infants before and after introduction of pneumococcal conjugate vaccine. JAMA 295:1668–1674 16609088 10.1001/jama.295.14.1668

[6] Centers for Disease Control and Prevention CDC 2008 Invasive pneumococcal disease in children 5 years after conjugate vaccine introduction—eight states, 1998–2005. MMWR Morb. Mortal. Wkly. Rep. 57:144–148 18272956

[7] Hicks LA Harrison LH Flannery B Hadler JL Schaffner W Craig AS Jackson D Thomas A Beall B Lynfield R Reingold A Farley MM Whitney CG 2007 Incidence of pneumococcal disease due to non-pneumococcal conjugate vaccine (PCV7) serotypes in the United States during the era of widespread PCV7 vaccination, 1998–2004. J. Infect. Dis. 196:1346–1354 17922399 10.1086/521626

[8] Singleton RJ Hennessy TW Bulkow LR Hammitt LL Zulz T Hurlburt DA Butler JC Rudolph K Parkinson A 2007 Invasive pneumococcal disease caused by nonvaccine serotypes among Alaska native children with high levels of 7-valent pneumococcal conjugate vaccine coverage. JAMA 297:1784–1792 17456820 10.1001/jama.297.16.1784

[9] Kim KH Hong JY Lee H Kwak GY Nam CH Lee SY Oh E Yu J Nahm MH Kang JH 2011 Nasopharyngeal pneumococcal carriage of children attending day care centers in Korea: comparison between children immunized with 7-valent pneumococcal conjugate vaccine and non-immunized. J. Korean Med. Sci. 26:184–190 21286007 10.3346/jkms.2011.26.2.184 PMC3031000

[10] Frazão N Sá-Leão R de Lencastre H 2010 Impact of a single dose of the 7-valent pneumococcal conjugate vaccine on colonization. Vaccine 28:3445–3452 20197144 10.1016/j.vaccine.2010.02.070

[11] Arrecubieta C López R García E 1994 Molecular characterization of *cap3A*, a gene from the operon required for the synthesis of the capsule of *Streptococcus pneumoniae* type 3: sequencing of mutations responsible for the unencapsulated phenotype and localization of the capsular cluster on the pneumococcal chromosome. J. Bacteriol. 176:6375–6383 7929009 10.1128/jb.176.20.6375-6383.1994 PMC196979

[12] Park IH Kim KH Andrade AL Briles DE McDaniel LS Nahm MH 2012 Nontypeable pneumococci can be divided into multiple *cps* types, including one type expressing the novel gene *pspK*. mBio 3(3):e00035-12. 10.1128/mBio.00035-12 22532557 PMC3340917

[13] Keller LE Jones CV Thornton JA Sanders ME Swiatlo E Nahm MH Park IH McDaniel LS 2013 PspK of *Streptococcus pneumoniae* increases adherence to epithelial cells and enhances nasopharyngeal colonization. Infect. Immun. 81:173–181 23115034 10.1128/IAI.00755-12 PMC3536125

[14] Angiuoli SV Gussman A Klimke W Cochrane G Field D Garrity G Kodira CD Kyrpides N Madupu R Markowitz V Tatusova T Thomson N White O 2008 Toward an online repository of standard operating procedures (SOPs) for (meta)genomic annotation. Omics 12:137–141 18416670 10.1089/omi.2008.0017 PMC3196215

